# Isolation and Biological Evaluation of Alfa-Mangostin as Potential Therapeutic Agents against Liver Fibrosis

**DOI:** 10.3390/bioengineering10091075

**Published:** 2023-09-11

**Authors:** Yi-Jen Liao, Chun-Ya Lee, Yuh-Ching Twu, Fat-Moon Suk, Tzu-Chieh Lai, Ya-Ching Chang, Yi-Cheng Lai, Jing-Wei Yuan, Hong-Ming Jhuang, Huei-Ruei Jian, Li-Chia Huang, Kuang-Po Chen, Ming-Hua Hsu

**Affiliations:** 1 School of Medical Laboratory Science and Biotechnology, College of Medical Science and Technology, Taipei Medical University, Taipei 110, Taiwan; yjliao@tmu.edu.tw (Y.-J.L.);; 2 Department of Biotechnology and Laboratory Science in Medicine, School of Biomedical Science and Engineering, National Yang Ming Chiao Tung University, Taipei 112, Taiwan; 3 Division of Gastroenterology, Department of Internal Medicine, Wan Fang Hospital, Taipei Medical University, Taipei 116, Taiwan; 4 Department of Internal Medicine, School of Medicine, College of Medicine, Taipei Medical University, Taipei 110, Taiwan; 5 Department of Chemistry, National Changhua University of Education, Changhua 500, Taiwan; 6 Department of Chemistry, Chinese Culture University, Taipei 111, Taiwan

**Keywords:** mangostin, alfa-mangostin, liver fibrosis

## Abstract

The increased proliferation and activation of hepatic stellate cells (HSCs) are associated with liver fibrosis development. To date, there are no FDA-approved drugs for the treatment of liver cirrhosis. Augmentation of HSCs apoptosis is one of the resolutions for liver fibrosis. In this study, we extracted α-mangostin (1,3,6-trihydroxy-7-methoxy-2,8-bis(3-methyl-2-butenyl)-9H-xanthen-9-one) from the fruit waste components of mangosteen pericarp. The isolated α-mangostin structure was determined and characterized with nuclear magnetic resonance (NMR) and high-resolution mass spectrometry (HRMS) and compared with those known compounds. The intracellular signaling pathway activities of α-mangostin on Transforming growth factors-beta 1 (TGF-β1) or Platelet-derived growth factor subunit B (PDGF-BB) induced HSCs activation and were analyzed via Western blot and Real-time Quantitative Polymerase Chain Reaction (Q-PCR). α-Mangostin-induced mitochondrial dysfunction and apoptosis in HSCs were measured by seahorse assay and caspase-dependent cleavage. The in vivo anti-fibrotic effect of α-mangostin was assessed by carbon tetrachloride (CCl_4_) treatment mouse model. The data showed that α-mangostin treatment inhibited TGF-β1-induced Smad2/3 phosphorylation and alpha-smooth muscle actin (α-SMA) expression in HSCs in a dose-dependent manner. Regarding the PDGF-BB-induced HSCs proliferation signaling pathways, α-mangostin pretreatment suppressed the phosphorylation of extracellular-signal-regulated kinase (ERK) and p38. The activation of caspase-dependent apoptosis and dysfunction of mitochondrial respiration (such as oxygen consumption rate, ATP production, and maximal respiratory capacity) were observed in α-mangostin-treated HSCs. The CCl_4_-induced liver fibrosis mouse model showed that the administration of α-mangostin significantly decreased the expression of the fibrosis markers (α-SMA, collagen-a2 (col1a2), desmin and matrix metalloproteinase-2 (MMP-2)) as well as attenuated hepatic collagen deposition and liver damage. In conclusion, this study demonstrates that α-mangostin attenuates the progression of liver fibrosis through inhibiting the proliferation of HSCs and triggering apoptosis signals. Thus, α-mangostin may be used as a potential novel therapeutic agent against liver fibrosis.

## 1. Introduction

**Figure 1:**
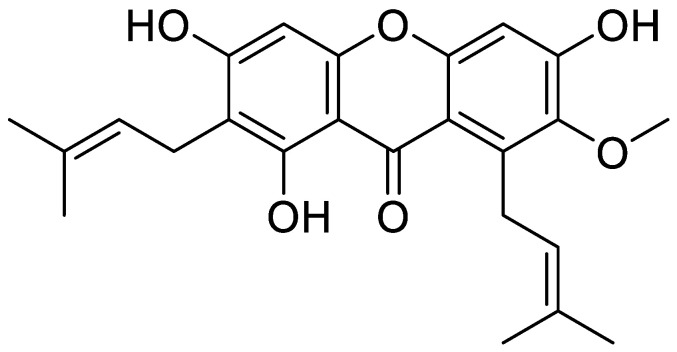
The structure of α-mangostin (1,3,6-trihydroxy-7-methoxy-2,8-bis(3-methyl-2-butenyl)-9H-xanthen-9-one).

α-Mangostin (1,3,6-trihydroxy-7-methoxy-2,8-bis(3-methyl-2-butenyl)-9H-xanthen-9-one) (Figure 1) is a xanthone derivative that is naturally found in the Garcinia mangostana L, a tropical fruit in Southeast Asia and is known as the “queen of fruits”. Recently, α-Mangostin has been considered as functional foods and supplements from the scientific community due to its potential health benefits [1,2]. It has been reported to exhibit a wide range of biological activities such as antioxidant [3], anti-inflammatory [4], anticancer [5,6], antimicrobial [7], antifungal [8], anti-vascular dementia [9], and neuroprotective properties [10]. Due to its unique xanthone core structure and diverse biological properties, α-Mangostin is considered to be a promising lead compound for the development of new drugs and therapeutic agents. However, its properties in ameliorating liver damage and liver fibrosis have not been studied yet. In this study, we present the pioneering discovery that α-Mangostin recovered from the mangosteen pericarp changes the balance of cell death over proliferation resulting in a remarkable anti-fibrotic effect on hepatic stellate cells.

Liver cirrhosis is a major cause of mortality and liver transplantation worldwide, and its therapeutic options are limited. In chronic liver diseases, regardless of their etiology (e.g., viral infection, alcohol abuse, or non-alcoholic fatty liver), the development of fibrosis is the first step toward the progression to cirrhosis, portal hypertension and hepatocellular carcinoma (HCC) [11,12,13]. Liver fibrosis is characterized by excessive extracellular matrix (ECM) deposition and fibrous scar formation [14]. Hepatic stellate cells (HSCs) are the major profibrogenic cells that produce ECM proteins in the damaged liver [15].

In response to multiple injurious agents or exposure to inflammatory cytokines, HSCs undergo an activation process from fat-storing quiescent phenotype to a myofibroblastic phenotype with significantly increased proliferation, chemotaxis, fibrogenesis, contractility, matrix degradation and retinoid loss [16,17,18,19]. Transforming growth factor-β1 (TGF-β1) is recognized as the key cytokine that activates HSCs and directly induces collagen 1 and α-smooth muscle actin (α-SMA) expression through Smad2/3 signal pathway, thus causing hepatic fibrogenesis and cancer progression [20,21,22]. TGF-β1 also stimulates the synthesis of extracellular matrix proteins and inhibits their degradation [23]. Antagonism of TGF-β1 signaling pathways markedly decreases fibrosis [24,25]. Apart from TGF-β1 signaling, HSCs activation induces the release of platelet-derived growth factor (PDGF), angiotensin II and vascular endothelial growth factor (VEGF), which are important for the sustained activation and proliferation of HSCs [16]. PDGF-BB, a highly HSCs mitogen, activates Ras and sequentially propagates a stimulatory signal via the phosphorylation of MAPK and Akt signaling pathways [20]. Blocking PDGF signaling ameliorates experimental liver fibrogenesis [26]. Inhibition of TGF-β1 or blocking Akt/P70S6K and ERK signaling pathways have been shown to attenuate HSCs activation [27,28]. Regarding inflammation, HSCs activation is also mediated by various pro-inflammatory cytokines released from the damaged hepatocytes or activated Kupffer cells. The events subsequent to HSCs activation, such as ECM production, promote proliferation and fibrogenesis [29,30]. Lipopolysaccharide (LPS) induces the secretion of chemokines/cytokines which can activate HSCs by triggering NF-κB and JNK phosphorylation through toll-like receptor 4 (TLR4) [31]. The activation of TLR4 signaling can enhance TGF-β signaling in HSCs [32].

Apoptosis is a major regulatory process governing the fate of hepatic myofibroblasts during the resolution phase [33,34,35]. Augmentation of HSCs apoptosis enhances the resolution of experimental liver fibrosis [36,37,38]; thereby highlighting that the fibrosis resolution pathway may represent a beneficial approach in terms of anti-fibrotic drug discovery. Until now, there are no FDA-approved drugs for the treatment of liver cirrhosis; therefore, there exists an urgent need to develop effective agents to treat liver fibrosis. Herein, we provide an exploration of the sustainable and eco-friendly aspect of utilizing tropical fruit waste, the mangosteen pericarp, for the extraction of α-mangostin. This innovative approach not only addresses environmental concerns by reducing fruit waste but also contributes to the development of economically viable treatment options for liver fibrosis. We found that α-mangostin not only suppressed HSCs proliferation and activation, but also triggered mitochondrial dysfunction and caspase-dependent apoptosis signal in HSCs. Administration of α-mangostin inhibited CCl_4_-induced liver fibrosis and damage in mice. 

## 2. Materials and Methods

### 2.1. Materials

All solvents used in the study were of high purity, either ACS or HPLC grade. Ethyl acetate, hexane and methanol were obtained from Macron Fine Chemicals (Taichung, Taiwan). The fruit hull of mangosteen (GML) was purchased from a local market. Analytical thin-layer chromatography (TLC) was performed on pre-coated plates (silica gel 60 F-254), purchased from Merck (Taipei, Taiwan). Column chromatography was performed on silica gel 60 (particle size 0.063–0.200 mm, 70–230 mesh ASTM). Nuclear magnetic resonance (1H NMR and ^13^C NMR) spectra were measured on a Bruker Avance 300 [300 MHz (^1^H), 75 MHz (^13^C)] spectrometer (Billerica, MA, USA). The chemical shifts are given in parts per million (ppm) on the delta (δ) scale. The solvent peak was used as a reference value, for ^1^H NMR: CDCl_3_ = 7.24 ppm, for ^13^C NMR: CDCl_3_ = 77.23 ppm.

### 2.2. Isolation and Purification of α-Mangostin from Mangosteen Pericarp

Mangosteen was purchased from the market, the pericarps were first cleaned with distilled water to remove impurities and then dried in an oven until all moisture was removed. Once dried, the pericarps were finely milled using a mortar and pestle, and the resulting powder was collected. To begin the extraction process, 5.0 g of the dried powder was subjected to maceration with 150 mL of methanol at room temperature for a duration of one day. The mixture was then filtered to separate the powder from the solution, and the filter solution was carefully collected. This maceration process was repeated three times, and each time, the filter solution was collected again. The next step involved concentrating the combined filter solutions obtained from the three repetitions. This was achieved using a rotary evaporator at a controlled temperature of 60 °C, resulting in the formation of a crude product with a brown, thick liquid consistency. Subsequently, the crude product was further purified through column chromatography. Elution was performed using an EtOAc/n-Hexane ratio of 50:50, successfully isolating the α-mangostin compound, the stander procedure for isolation of α-mangostin were in the Supplementary Materials Figure S1. The structures of the isolated compounds were determined with nuclear magnetic resonance (Figure S2 in the Supplementary Materials) and compared with standard compounds (Figure S3 in the Supplementary Materials) and those of known compounds reported in the literature. The purity of the compounds was confirmed by high-performance liquid chromatography (HPLC) to be higher than 98%.

### 2.3. Cell Culture

HSC-T6 cells [39] were cultured in Dulbecco’s modified Eagle’s medium (Gibco BRL, Grand Island, NY, USA) with 1% heat-inactivated fetal bovine serum (HyClone, Logan, UT, USA), penicillin (100 U/mL), streptomycin (100 μg/mL), nonessential amino acids (0.1 mM), and L-glutamine (2 mM) in a humidified incubator with 5% CO_2_.

### 2.4. TGF-β1, LPS, and PDGF-BB Treatments

To evaluate effects of α-mangostin on HSCs activation, HSC-T6 cells were treated with 10 ng/mL TGF-β1 (R&D Systems, Minneapolis, MN, USA) in the presence or absence of α-mangostin for indicated hours and then subjected to real-time PCR and Western blot analyses. For intracellular signaling pathways analyses, HSC-T6 cells were pretreated with 5µM α-mangostin overnight, followed by stimulation with TGF-β1 (10 ng/mL), PDGF-BB (10 ng/mL) or LPS (100 ng/mL) for different time periods and then subjected to Western blot analyses.

### 2.5. Cell Viability Assay

For viability assays, cells were seeded in a 96-well plate. After the indicated treatments, cell viability was measured by commercial alamar blue assay kit (Life Technologies, Carlsbad, CA, USA). Subsequently, 10 µL alamarBlue^®^ reagent was added to 100 µL culture media. Incubation was conducted for 2.5 h at 37 °C in a cell culture incubator, protected from direct light. The absorbance of alamarBlue^®^ was monitored at 570 nm, using 600 nm as a reference wavelength (normalized to the 600 nm value). The survival percentages were calculated by dividing the OD value of treatment groups by solvent control group.

### 2.6. Animals and Experimental Design

Eight-week-old C57BL/6 male wild-type (WT) mice were purchased from the National Laboratory Animal Center, Taiwan. All mice were maintained on a standard chow diet (no. 5001, LabDiet, St. Louis, MO, USA) and housed in a 12-h/12-h light/dark cycle. The mice were assigned randomly to three groups: (1) vehicle control, (2) CCl_4_ (2 mL/kg body weight [1:5 *v*/*v* in corn oil]) intraperitoneal injection twice weekly for 7 weeks, and (3) For therapeutic evaluation, CCl_4_ (2 mL/kg body weight [1:5 *v*/*v* in corn oil]) intraperitoneal injection twice weekly for 2 weeks to induce liver fibrosis, and then administrated α-mangostin (5 mg/kg daily intraperitoneal injection) concomitantly with CCl_4_ injection for another 5 weeks. The experimental protocols were approved by the Institutional Animal Care and Use Committee of Taipei Medical University. The tissues used in protein and RNA analyses were frozen in liquid nitrogen and stored at −80 °C before use, while those used in IHC staining were fixed in 10% formalin.

### 2.7. Western Blot and IHC Staining

Cultured cells and liver tissues were lysed using lysis buffer supplemented with protease and phosphatase inhibitors. Proteins (30 µg) were separated by SDS-PAGE. Rabbit anti-p-Smad2/3, T-Smad2/3, p-MEK, T-MEK, p-ERK, T-ERK, p-JNK, T-JNK, p-p38, T-p38, Caspase-8, Caspase-3, Caspase-9, and PARP were purchased from Cell Signaling Technology (Beverly, MA, USA). Rabbit anti-α-SMA was purchased from Abcam (Cambridge, MA, USA). The immunoblotting signals were normalized to that for α-tubulin (Sigma-Aldrich, St. Louis, MO, USA). Sirius red staining (Abcam) of paraffin-embedded liver sections was used to qualitatively assess the collagen architecture and the extent of fibrosis in accordance with the manufacturer’s instructions.

### 2.8. Real-Time PCR

Total RNA was isolated from mouse liver using TRIzol Reagent (Ambion, Carlsbad, CA, USA), according to the manufacturer’s protocol. Complementary DNA was produced from cellular RNA (2 μg) using a High Capacity RNase H-Reverse Transcriptase Kit (Invitrogen, Carlsbad, CA, USA). The following primers were used in the real-time PCR (Q-PCR): α-SMA, 5′-GTTCAGTGGTGCCTCTGTCA-3′ (forward) and 5′-ACTGGGACGACATGGAAAAG-3′ (reverse); collagen type 1 alpha 2 (col1A2): 5′-TAGGCCATTGTGTATGCAGC-3′ (forward) and 5′-ACATGTTCAGCTTTGTGGACC-3′ (reverse); MMP2, 5′-CTCAGATCCGTGGTGAGAT-3′ (forward) and 5′-AGGCTGGTCAGTGGCTTGG-3′ (reverse); TGF-β: 5′-CGAAGCGGACTACTATGC-3′ (forward) and 5′-GTTGCTCCACACTTGATTT-3′ (reverse); desmin: 5′-CAGGCAGCCAATAAGAAC-3′ (forward) and 5′-GCCATCTCATCCTTAGGT-3′ (reverse); glyceraldehyde-3-phosphate dehydrogenase (GAPDH): 5′-TCACCACCATGGAGAAGGC-3′ (forward); and 5′-GCTAAGCAGTTGGTGGTGCA-3′ (reverse). Reactions (10 μL) were contained 4 µL template cDNA (20 ng), 5 µL KAPA SYBR^®^ FAST qPCR Master Mix (2X), and 1 µL forward/reverse primer mix (6 µM each) (KAPA Biosystems, Boston, MA, USA). Thermal cycling consisted of 15 min at 95 °C, followed by 40 cycles at 95 °C for 15 s and 60 °C for 60 s using the StepOne System (AppliedBiosystems Waltham, MA, USA). The predicted cycle threshold (CT) values were exported into Excel worksheets for analysis. Comparative CT methods were used to determine the gene expression levels relative to that for GAPDH.

### 2.9. Seahorse Assay

In vivo cell real-time cellular oxygen consumption rate (OCR) was measured by an XF24 bioenergetic assay (Seahorse Bioscience, Billerica, MA, USA), according to the manufacturer’s instructions. Briefly, HSC-T6 cells were seeded in a XF24-well plate containing complete medium. After 16 h, the XF24 bioenergetic assay was initiated by removing the exhausted medium and replacing it with sodium-bicarbonate-free DMEM containing 2% FBS. The OCR was detected at a steady state, then oligomycin (1 µM), carbonyl cyanide 4-[trifluoromethoxy] phenylhydrazone (FCCP; 2 μM), Rotenone/Antimycin A (AA; 0.5 μM) were injected sequentially into the wells to obtain the values of the maximal and non-mitochondrial respiration rate.

### 2.10. Statistical Analyses

In vitro data are shown as mean ± standard deviation (SD). In vivo data are shown as mean ± standard error of the mean (sem). Statistical analyses were performed using the SPSS program (version 13, SPSS, Chicago, IL, USA). At least three independent experiments were carried out for each sample. Either ANOVA or Student’s *t*-test was used for group comparisons, with a probability value of *p* < 0.05 considered to indicate statistical significance.

## 3. Results

### 3.1. α-Mangostin Inhibits TGF-β1-Induced HSCs Activation

**Figure 2:**
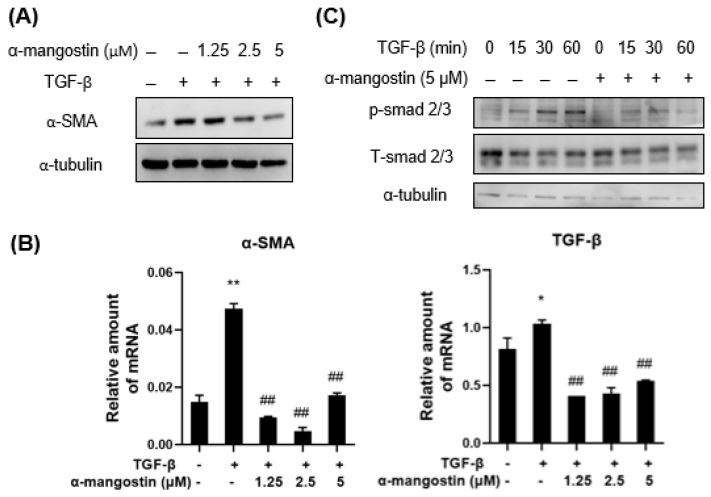
α-mangostin inhibits TGF-β1-induced HSCs activation. HSC-T6 cells were treated with TGF-β1 (10 ng/mL) and α-mangostin for 24 h. (**A**) Western blot analysis of protein levels of α-SMA and α-tubulin. (**B**) Real-time PCR of mRNA levels of α-SMA and TGF-β relative to that of GAPDH. (**C**) HSC-T6 cells were pre-treated with α-mangostin (5 µM) overnight and then subjected to TGF-β1 (10 ng/mL) for the indicated times. Phosphorylation- and total-smad2/3 were analyzed by Western blot. *, *p* < 0.05 vs. control group; **, *p* < 0.01 vs. control group; ##, *p* < 0.01 vs. TGF-β1 alone group.

Activated HSCs that express α-SMA are responsible for liver fibrosis due to the deposition of excessive ECM and increase in collagen accumulation mediated by TGF-β1 [20,22]. Therefore, we first determined whether α-mangostin suppresses the activation of HSCs. WB analysis showed that the TGF-β1-induced α-SMA upregulation was decreased in α-mangostin-treated HSC-T6 cells in a dose-dependent manner (Figure 2A). Q-PCR analysis showed that α-mangostin treatment significantly reduced the mRNA levels of α-SMA and TGF-β in HSCs (Figure 2B). Once TGF-β1 binds to its receptor, Smad2/3 protein becomes phosphorylated and then triggers collagen production [16,19]. Therefore, we tested whether α-mangostin treatment inhibits smad2/3 activation in TGF-β1-treated HSCs. As shown in Figure 2C, α-mangostin treatment reduced the phosphorylation of Smad2/3 relative to the control.

### 3.2. α-Mangostin Inhibits HSCs Proliferation through Blocking PDGF-BB-Induced ERK and p38 Signaling

**Figure 3:**
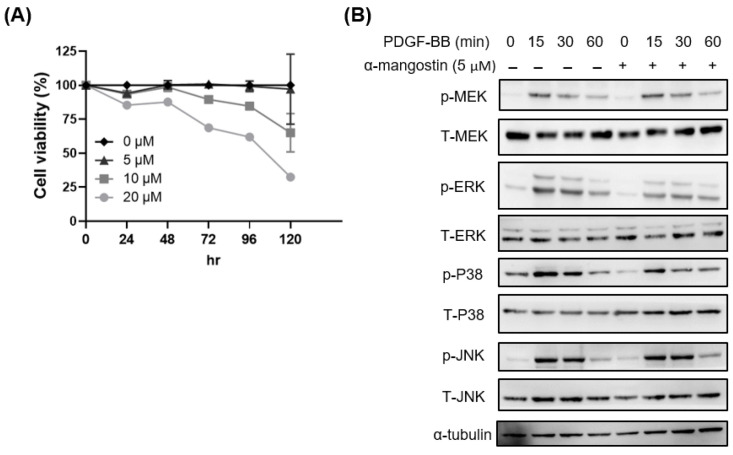
α-mangostin inhibits HSCs proliferation via inhibition of ERK/p38 activation. (**A**) HSC-T6 cells were treated with 0, 5, 10, 20 μM of α-mangostin for indicated hours and then subjected to alamar blue analysis. (**B**) HSC-T6 cells were pre-treated with α-mangostin (5 µM) overnight and then subjected to PDGF-BB (10 ng/mL) for the indicated mins. Phosphorylation and total indicated proteins were analyzed by Western blot.

Activation of HSCs into proliferation is the critical stage to promote liver fibrogenesis [16]. To evaluate the effects of α-mangostin on the proliferation of HSCs, 0–20 µM of α-mangostin were treated the HSC-T6 cells for 24~120 h. The dose-dependent and time-dependent inhibitions of HSC-T6 cells proliferation were observed after α-mangostin treatment (Figure 3A). Since PDGF-BB has been identified as the most potent stimuli for proliferation of HSCs [19], we next examined the effects of α-mangostin on PDGF-BB stimulated intracellular pathway in HSC-T6 cells, and focused on MAPK pathway. As indicated in Figure 3B, α-mangostin pretreatment decreased PDGF-BB-induced ERK and p38 phosphorylation. However, the phosphorylation of MEK and JNK were not changed.

### 3.3. α-Mangostin Induces Apoptosis in HSCs

**Figure 4:**
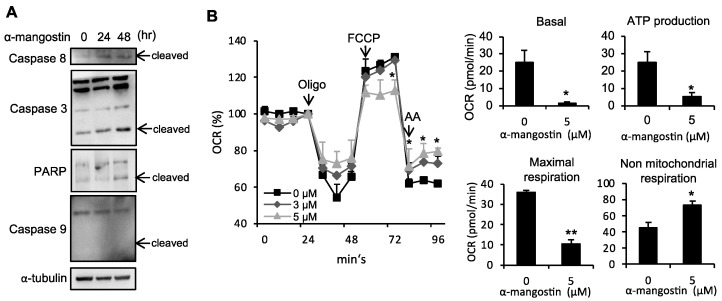
α-mangostin induces mitochondrial dysfunction and apoptosis in HSCs. (**A**) Protein levels of Caspase-8, 3, 9 and PARP in α-mangostin (3 µM) treated HSC-T6 cells were analyzed using Western blot. (**B**) HSC-T6 cells were pre-treated with α-mangostin x Analyzer. Oligomycin (1 μM), FCCP (2 μM) and rotenene/antimycin (AA, 0.5 μM) were injected into the well at the 4th, 8th and 11th time points. Quantitative data are shown in right panel. *, *p* < 0.05 vs. control group; **, *p* < 0.01 vs. control group.

Next, we examined whether α-mangostin inhibited cell growth through inducing apoptosis signaling. As shown in Figure 4A, α-mangostin treatment promotes cleavage of caspase-8, 3, and PARP in a dose-dependent manner. However, there is no effect on caspase-9 cleavage. Since mitochondria play a key role in cell metabolism [40] which control cell growth and death [41], we next examined whether α-mangostin affects mitochondrial function in HSCs. Figure 4B shows that the basal respiration of mitochondria was decreased in α-mangostin-treated HSC-T6 cells. In addition, α-mangostin-treated cells showed less oxygen participated in ATP production. FCCP-induced maximal respiratory capacity was also decreased in α-mangostin-treated cells. Non-mitochondrial respiration was enhanced in α-mangostin-treated cells. These data suggest that the suppression of HSCs proliferation of α-mangostin arises primarily from inhibiting the ERK and p38 phosphorylation, as well as inducing the mitochondrial dysfunction and apoptosis.

### 3.4. α-Mangostin Ameliorates CCl_4_-Induced Liver Fibrosis in Mice

**Figure 5:**
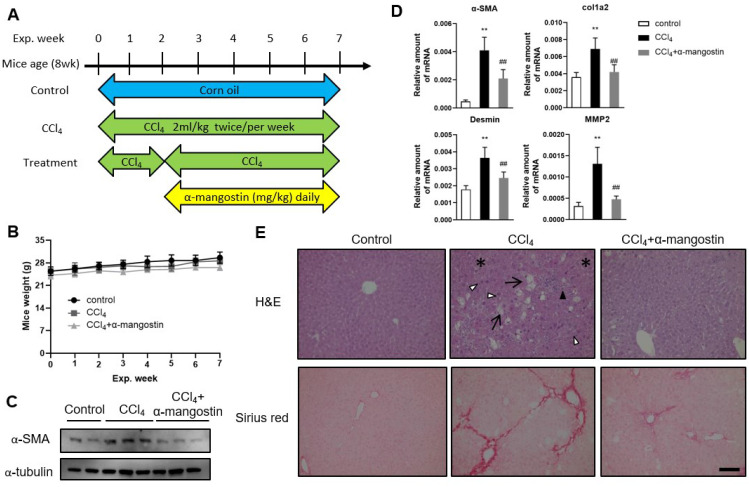
α-mangostin treatment attenuates CCl_4_-induced liver damage and fibrosis in mice. (**A**) Experimental scheme of liver fibrosis mice treated with α-mangostin. Eight-week-old C57BL/6 male mice received ip injection with oil (vehicle control), CCl_4_ (2 mL/kg, twice weekly for 7 weeks), or CCl_4_ (2 mL/kg, twice weekly for 2 weeks to induce liver fibrosis), and then administrated α-mangostin (5 mg/kg, daily) concomitantly with CCl_4_ injection for another 5 weeks. (**B**) Mice weights were measured per week. (**C**) Western blot analysis of hepatic protein levels of α-SMA and α-tubulin from CCl_4_ and CCl_4_+α-mangostin treated mice. (**D**) Real-time PCR analyzed the mRNA levels of α-SMA, col1a2, MMP2 and desmin relative to that of GAPDH (*n* = 5 per group). The data are expressed as the mean ± sem. (**E**) Representative images of H&E and Sirius red staining of liver tissues from each treatment group. Arrows, fatty change; white triangles, perinuclear vacuoles; black triangles, regional inflammation; asterisks, irregular infiltration borders. Scale bar = 200 μm; **, *p* < 0.01 vs. control group; ##, *p* < 0.01 vs. CCl_4_ alone group.

To further evaluate the therapeutic anti-fibrotic efficacy of α-mangostin, we utilized a widely used experimental mouse model of liver fibrosis induced by CCl_4_ injections (Figure 5A). For the treatment group, the mice received CCl_4_ two weeks to induce liver fibrosis, and then administrated α-mangostin concomitantly with CCl_4_ injection for another five weeks. Mice were weighed weekly to check for side effects associated with α-mangostin. The weight changes within three groups during the seven-week period showed no significant difference (Figure 5B). As shown in Figure 5C, α-mangostin treatment reduced CCl_4_-induced hepatic α-SMA expression. The inhibitory effect of α-mangostin on liver fibrosis markers was also demonstrated by the mRNA levels of α-SMA, col1a2, MMP2 and desmin (Figure 5D). Regarding the hepatic pathological examination by H&E staining, CCl_4_ induced significant irregular infiltration borders perinuclear vacuoles, fatty change and regional inflammation in the liver (Figure 5E). Meanwhile, normal appearance was found in α-mangostin-treated groups (Figure 5E). Collagen deposition, a marker for liver fibrosis, was assessed by Sirius red staining. A large amount of collagen deposition was observed in CCl_4_-treated mice, whereas this phenomenon was attenuated in α-mangostin-treated mice (Figure 5E). These data indicate that α-mangostin administration attenuates liver fibrosis and liver damage in CCl_4_-treated mouse model.

## 4. Discussion

We can obtain pure α-mangostin from the waste components of mangosteen pericarp with a yield of 5% by weight. Herein, we provide an exploration of the hidden potential of tropical fruit waste components, with a particular focus on the compound α-mangostin derived from the mangosteen pericarp, as a valuable and promising remedy for liver fibrosis. Liver fibrosis is a common consequence of various chronic liver diseases, and its progression can lead to severe complications, including liver cirrhosis and hepatocellular carcinoma.

**Figure 6:**
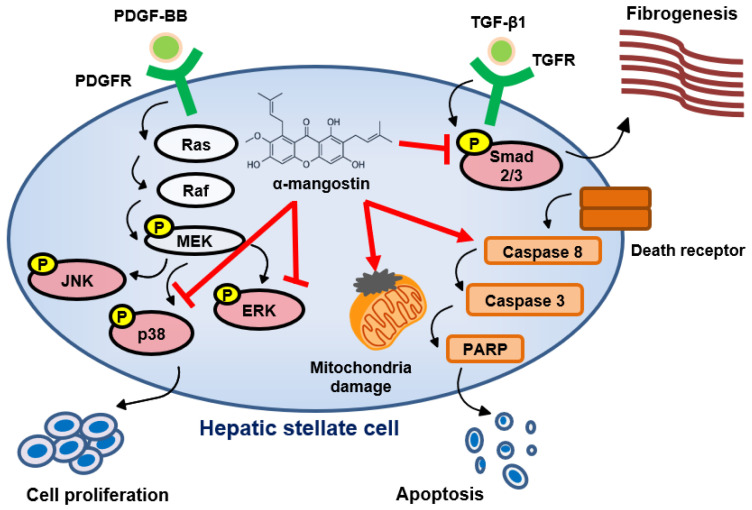
Proposed model of α-mangostin in the inhibition of liver fibrogenesis.

Cirrhosis is a global health problem, which is associated with substantial economic burden [42]. Therefore, the prevention of fibrosis progression is critical to improve health-associated quality of life in patients and decreases the likelihood of subsequent cirrhosis and liver cancer development. In our study, we found that α-mangostin displays anti-fibrotic properties in vitro and in vivo. As shown in Figure 6, α-mangostin significantly inhibits HSCs activation and proliferation through blocking smad2/3 and ERK/p38 phosphorylation. Compound α-mangostin also triggers mitochondrial dysfunction and caspase-dependent apoptosis signaling in HSCs. Finally, we proved that α-mangostin administration can ameliorate CCl_4_-induced liver fibrosis and damage in mice.

Inhibition of HSCs proliferation, activation, and induction of HSCs apoptosis are the therapeutic targets in liver fibrosis [24,33]. The TGF-β/Smad signaling pathway plays a critical role in the initiation of liver fibrogenesis. Upon TGF-β1 binding to its receptors, Smad2/3 proteins are phosphorylated and form a hetero-oligomeric complex with Smad4. The complex then translocates into the nucleus of HSCs where it induces collagen genes transcription and mediates hepatic fibrosis [43]. Our study revealed that α-mangostin selectively decreased TGF-β1-induced Smad2/3 phosphorylation and α-SMA expression (Figure 2). PDGF-BB, a major stimulator of HSCs proliferation, activates MAPK and Akt signaling during liver fibrogenesis [26]. The activation of MAPK/ERK, PI3K/Akt and its downstream molecular p70S6K pathway is involved in cell growth, differentiation, and protein synthesis of HSCs [44,45,46]. Inhibition of either the MAPK or PI3K/Akt pathways suppress HSCs activation and liver fibrosis in mice [47,48]. In the present study, we showed that α-mangostin recovered from fruit waste components of mangosteen pericarp, expresses its beneficial effects on suppression of both ERK and p38 phosphorylation in PDGF-BB-treated HSCs (Figure 3).

Apoptosis can occur either through mitochondria/caspase system or through the death-receptor pathways [49]. Augmentation of HSCs apoptosis using pharmacological approach has been shown to enhance the resolution of liver fibrosis [37,38,50]. In this study, we showed that α-mangostin treatment can decrease mitochondrial respiration functions and trigger the cleavage of caspase cascade in HSCs (Figure 4), thus, resulting in HSCs apoptosis. Cell death programs are discovered in several pathways such as apoptosis, autophagy, ferroptosis, etc. Ferroptosis is an iron-dependent and non-apoptotic cell death which is activated by toxic lipid reactive oxygen species and is said to be a potential mechanism to treat fibrosis [51]. Kong et al. found that artesunate induced ferroptosis and alleviated HSCs activation [52]. However, ferroptosis somehow acts as a two-edged sword in fibrosis development; therefore, further studies are required to elaborate the interaction between ferroptosis and fibrosis.

The inhibition of HSCs activation and proliferation is considered to be an effective approach for liver fibrosis treatment. Several anti-fibrotic drugs, which promote anti-inflammatory, anti-oxidation, ECM degradation, and TGF-β signaling pathway, are under clinical trials [53]. A recent study found that the combination of Saikosaponin b1 nanodrugs and antioxidant MnO_2_ removed excessive H_2_O_2_ and reduced hypoxia-induced TGF-β1, ultimately treating liver fibrosis [54]. On top of that, these studies indicate the multifaceted role of anti-fibrotic treatment. In our study, α-mangostin displayed anti-fibrotic effects through inhibiting the TGF-β/PDGF-BB signaling pathway and inducing HSCs apoptosis, providing a double-effect on fibrosis treatment. Additionally, a new drug delivery system for α-mangostin should be considered in future applications for antifibrosis treatment. A novel nanotechnology can be a potential drug delivery system for α-mangostin to improve solubility and affect pharmacokinetic and pharmacodynamic aspects. Nanoparticles, nanomicelles, and liposome nanoparticles can present a promising strategy for the delivery of α-mangostin in antifibrosis treatment [55].

## 5. Conclusions

In conclusion, our study revealed that α-mangostin not only suppressed both TGF-β1 and PDGF-BB-induced phosphorylation of Smad2/3, ERK and p38, but also triggered mitochondrial dysfunction and caspase-dependent apoptosis signaling in HSCs. Furthermore, α-mangostin administration significantly ameliorates CCl_4_-induced liver damage and fibrosis in mice. Thus, this novel compound changes the balance of cell death over proliferation exhibiting significant anti-fibrotic activity in HSCs, which suggests its potential use as a novel therapeutic strategy for patients with liver fibrosis.

## Data Availability

Requests for resources and reagents should be contacted to the corresponding author.

## Supplementary Materials

The following supporting information can be downloaded at: https://www.mdpi.com/article/10.3390/bioengineering10091075/s1, Figure S1, Stand procedure for isolation of α-mangostin; Figure S2, the NMR spectra of isolated α-mangostin; Figure S3, α-mangostin standard NMR spectra.
