# Supplementary material for: Isolation and Biological Evaluation of Alfa-Mangostin as Potential Therapeutic Agents against Liver Fibrosis
Source: Bioengineering (Basel). 2023 Sep 11;10(9):1075. doi: 10.3390/bioengineering10091075 (PMC10526009; doi:10.3390/bioengineering10091075)
Supplement: Supplementary file 1 [file bioengineering-10-01075-s001.zip › bioengineering-2553811-supplementary.pdf]

Supplementary Materials

Isolation and Biological Evaluation of Alfa-Mangostin as Potential Therapeutic Agents against Liver Fibrosis

S1. Standard Procedure for Isolation of α-Mangostin

- The pericarps were first cleaned with distilled water to remove impurities and then dried in an oven until all moisture was removed—(Figure S1A)
- Once dried, the pericarps were finely milled using a mortar and pestle, and the resulting powder was collected—(Figure S1B, Figure S1C)
- To begin the extraction process, 5.0 g of the dried powder was subjected to maceration with 150 mL of methanol at room temperature for a duration of one day. The mixture was then filtered to separate the powder from the solution, and the filter solution was carefully collected. This maceration process was repeated three times, and each time, the filter solution was collected again—(Figure S1D)
- The combined filter solutions obtained from the three repetitions were concentrated using a rotary evaporator at a controlled temperature of 60 °C. The process resulted in the formation of a crude product with a brown, thick liquid consistency—(Figure S1E)
- The crude product was further purified through column chromatography. Elution was performed using an EtOAc/n-Hexane ratio of 50:50, successfully isolating the α-mangostin compound—(Figure S1F)

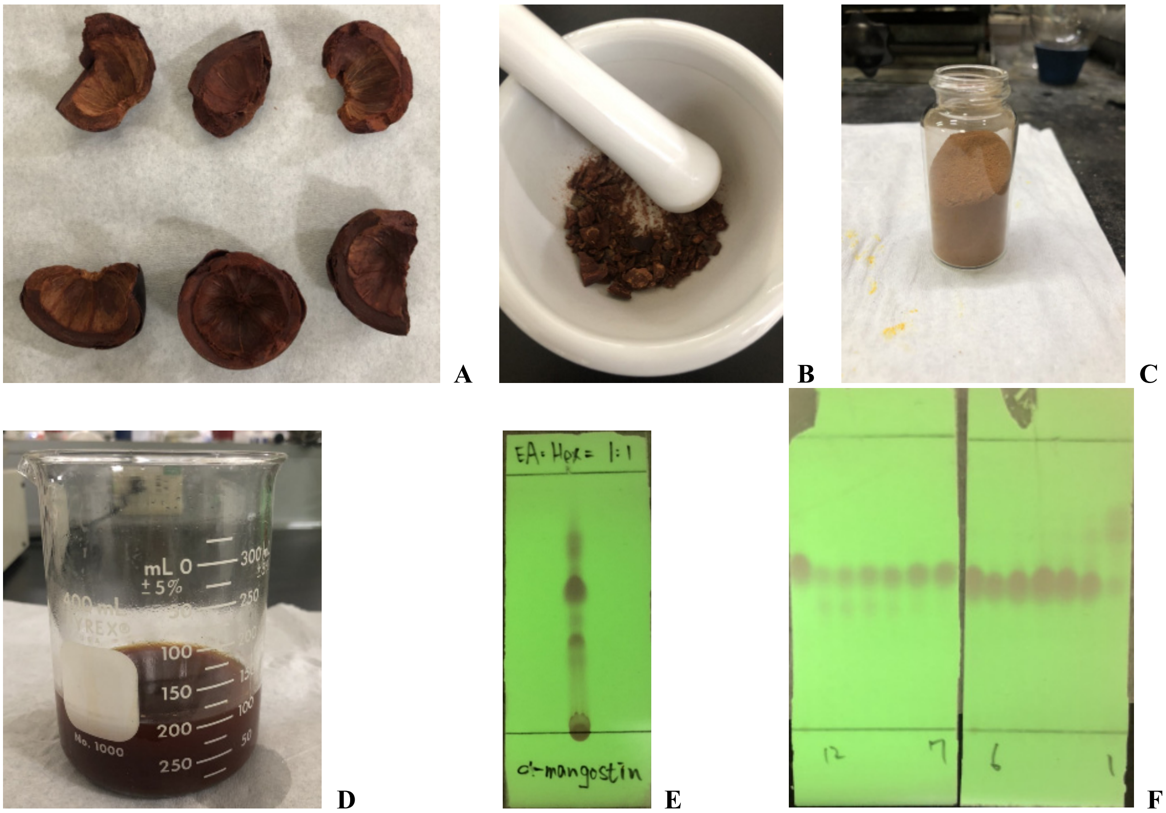

Figure S1. Standard Procedure for Isolation of α-Mangostin.

S2. Nuclear Magnetic Resonance (NMR)

Nuclear magnetic resonance (1H NMR and 13C NMR) spectra were measured on a Bruker Avance 300 [300 MHz (1H), 75 MHz (13C)] spectrometer. The chemical shifts are given in parts per million (ppm) on the delta (δ) scale. The solvent peak was used as a reference value, for 1H NMR: CDCl3 = 7.24 ppm, for 13C NMR: CDCl3 = 77.23 ppm.

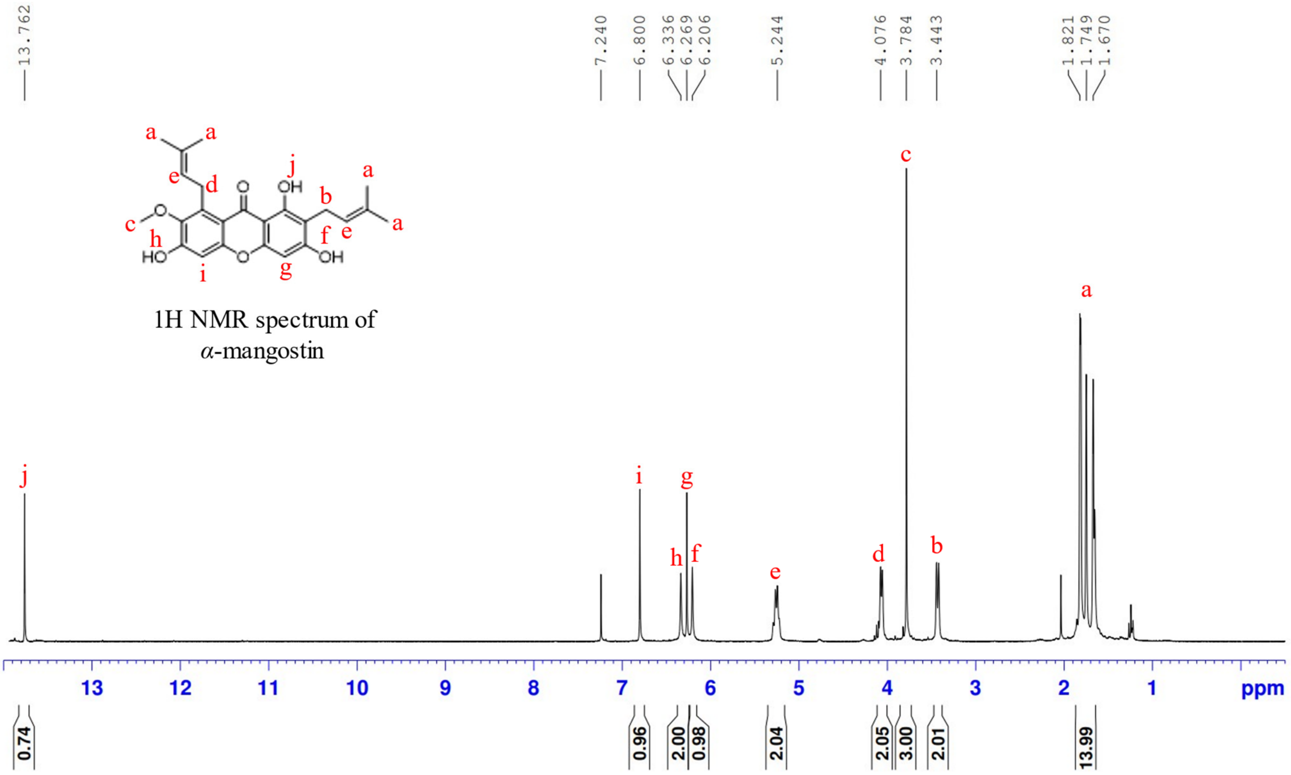

Figure S2. Isolated α-mangostin NMR spectra

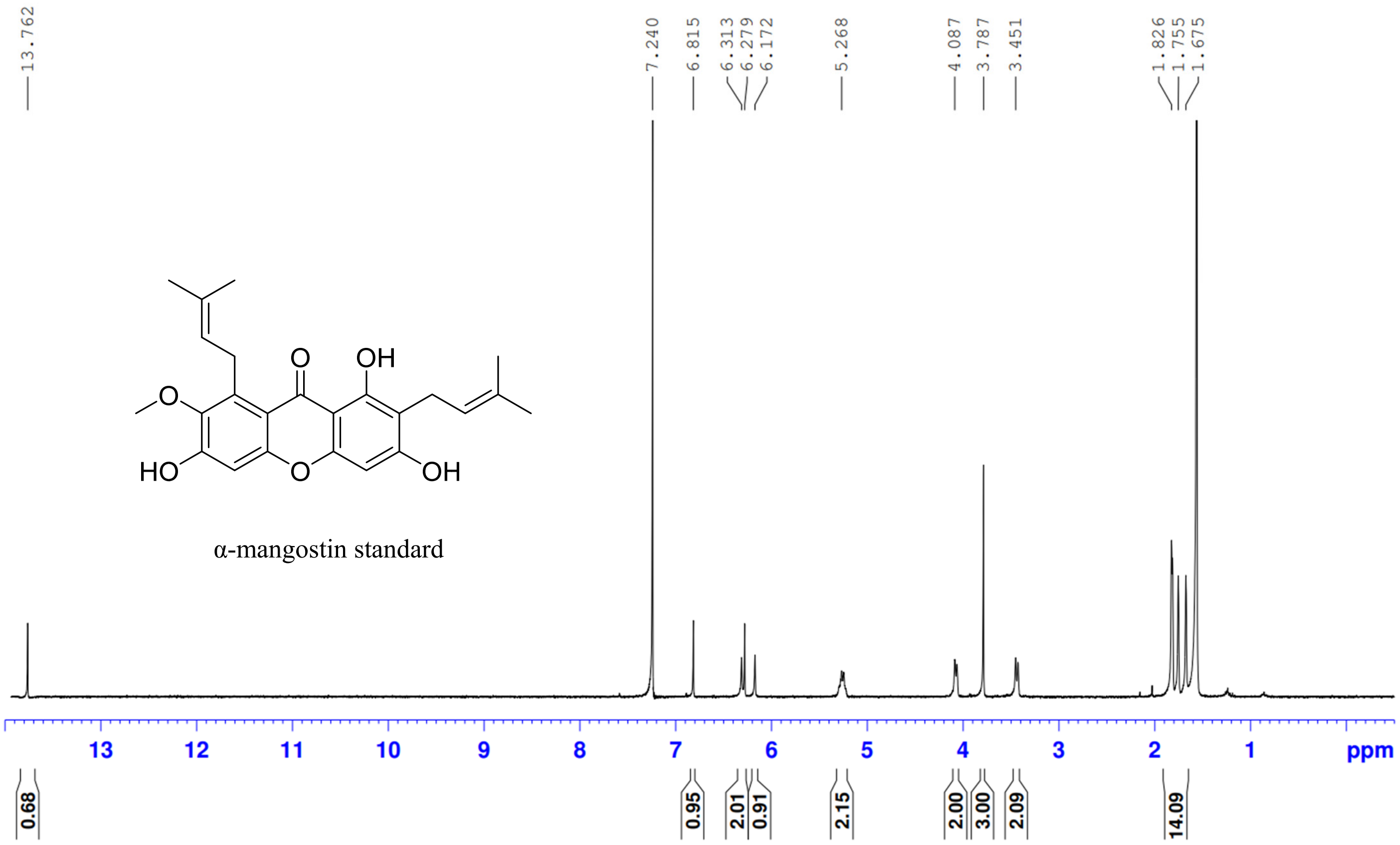

Figure S3. α-mangostin standard spectra
